# The Prevalence, Etiological Agents, Clinical Features, Treatment, and Diagnosis of HIV-Associated Oral Candidiasis in Pediatrics Across the World: A Systematic Review and Meta-Analysis

**DOI:** 10.3389/fped.2021.805527

**Published:** 2021-12-24

**Authors:** Zahra Rafat, Elahe Sasani, Yahya Salimi, Samaneh Hajimohammadi, Mohammad Shenagari, Davoud Roostaei

**Affiliations:** ^1^Department of Medical Microbiology, School of Medicine, Guilan University of Medical Sciences, Rasht, Iran; ^2^Department of Medical Mycology, Faculty of Medical Sciences, Tarbiat Modares University, Tehran, Iran; ^3^Social Development & Health Promotion Research Center, Health Institute, Kermanshah University of Medical Sciences, Kermanshah, Iran; ^4^Department of Toxicology, Faculty of Medical Sciences, Tarbiat Modares University, Tehran, Iran; ^5^Cellular and Molecular Research Center, School of Medicine, Guilan University of Medical Sciences, Rasht, Iran; ^6^Department of Pharmacology, School of Medicine, Guilan University of Medical Sciences, Rasht, Iran

**Keywords:** HIV, aids, pediatrics, children, infants, oral candidiasis, prevalence, meta-analysis

## Abstract

In HIV-infected pediatrics, oral candidiasis (OC) is a global issue of concern due to its association with dysphagia, malnutrition, and mortality. The present systematic review and meta-analysis are the first to determine the prevalence of OC in HIV-infected pediatrics worldwide. We searched international (PubMed, Web of Science, Scopus, and Embase) databases for studies published between January 2000 to May 2020 reporting the epidemiologic features of OC in HIV-infected pediatrics. Inclusion and exclusion criteria were defined to select eligible studies. Data were extracted and presented according to PRISMA guidelines. The results of the meta-analysis were visualized as a forest plot. Heterogeneity was also analyzed using the *I*^2^, and τ^2^ statistics. The publication bias was evaluated using Egger test. The literature search revealed 1926 studies, of which 34 studies met the eligibility criteria, consisting of 4,474 HIV-infected pediatrics from 12 different countries. The overall prevalence of OC among HIV-infected pediatrics was 23.9% (95% CI 17.3–32.0%), and *Candida albicans* was the most prevalent etiologic agent. Pseudomembranous candidiasis was the predominant clinical manifestation in HIV-infected pediatrics suffering from OC. Thirty articles involving 4,051 individuals provided data on HIV treatment status. Among the 4,051 individuals, 468 (11.53%) did not receive HIV treatment. The data from 11 articles demonstrated that HIV treatment was significantly associated with a reduction in oral *Candida* colonization or infection. In contrast, others showed the opposite relationship or did not report any statistical data. A high level of *I*^2^ (*I*^2^ = 96%, *P* < 0.01) and τ^2^ (τ^2^ = 1.36, *P* < 0.01) was obtained among studies, which provides evidence of notable heterogeneity between studies. OC is approximately frequent in HIV-positive children. Therefore, efforts should be made to teach dental and non-dental clinicians who care for HIV-infected pediatrics to diagnose and treat this infection.

## Introduction

*Candida* species reside as part of the normal flora of the oral cavity in about 66% of the pediatric population known as carriers (1). In the event of immunosuppression, especially in acquired immunodeficiency syndrome (AIDS), there is a shift from commensalisms to an exponential increase in colonization, which eventually leads to clinical signs and symptoms of oral candidiasis (OC) (2, 3). CD4 lymphocytes have a major role in the suppression of OC, and as a result, AIDS patients with low CD4 lymphocyte counts are especially susceptible to *Candida* infections (4). OC occurs in up to 95% of human immunodeficiency virus (HIV)-infected individuals during their illness and often represents the first form in which immunosuppression shows itself (5). According to the Joint United Nations Programme on HIV/AIDS (UNAIDS)-2020, there were 38.0 million people living with HIV in 2019, and 1.8 million of whom were pediatrics (6). The majority of studies conducted worldwide agree that OC is the most common HIV-related oral lesion; both in patients undergoing highly active antiretroviral therapy (HAART) and in untreated ones (7). There is some evidence that candidal infection may induce immunosuppression. Thus, candidiasis may enhance the progression to more severe disease and AIDS (8).

In recent years, the number of HIV-infected infants and children has grown remarkably, with more than 230% increase from 2010 (3). In 2017, an estimated 180,000 children ages 0–14 years were newly infected with HIV (9). The development of HIV infection in children has different characteristics to those noted in adults, due mainly to the immaturity of the immunologic system, and other body structures (10). In children, diseases usually progress faster, and outcomes are more serious than adults, resulting in a high mortality rate due to opportunistic infections like candidiasis (11).

The majority of oral *Candida* infections in HIV-infected patients are caused by *Candida albicans*, although, recently, an increasing number of infections have been associated with other *Candida* species which are more resistant to azoles and had created a public health concern (12–14). Some of the changes in *Candida* species prevalence have been associated with the increased use of antifungal agents for both prophylactic and treatment purposes (15). The common non-*Candida albicans* species associated with OC include *Candida glabrata, Candida parapsilosis, Candida tropicalis*, and *Candida krusei (Issatchenkia orientalis*) (15, 16).

OC is an important part of the clinical picture of HIV infection. Children with OC manifest discomfort during eating; this situation may reduce their food intake. Malnutrition is an important mortality risk factor in HIV-infected children because it reduces the capacity of the body to fight this infection by compromising various immune parameters (17).

Furthermore, OC has been attributed with important diagnostic and prognostic values to HIV infection and as a marker of antiretroviral therapy failure and, therefore can be used as a criterion to make important therapeutic decision-making (18).

Several articles have investigated the prevalence of OC in HIV-infected pediatric patients in different countries, but until now there is no comprehensive estimation of the infection among HIV-infected pediatric patients across the world. Therefore, performing a systematic review and meta-analysis was necessary to provide a more in-depth understanding and summarized evidence relating to OC in HIV-infected children and assist in its control.

## Materials and Methods

### Research Question

This study aims to determine the prevalence, the main etiologic agent, the predominant clinical manifestation, and the demographic characteristics of OC (colonization and/or infection) among HIV-infected pediatrics across the world.

### Research Strategy

This systematic review and meta-analysis was conducted based on PRISMA (Preferred Reporting Items for Systematic Reviews and Meta-Analyses) guidelines (19). We searched English databases (PubMed, Web of Science, Scopus, and Embase) for articles regarding the prevalence of oral *Candida* colonization or infection in HIV-infected pediatric patients published from January 2000 to May 2020. We also used references of included primary articles for search.

**Figure 1:**
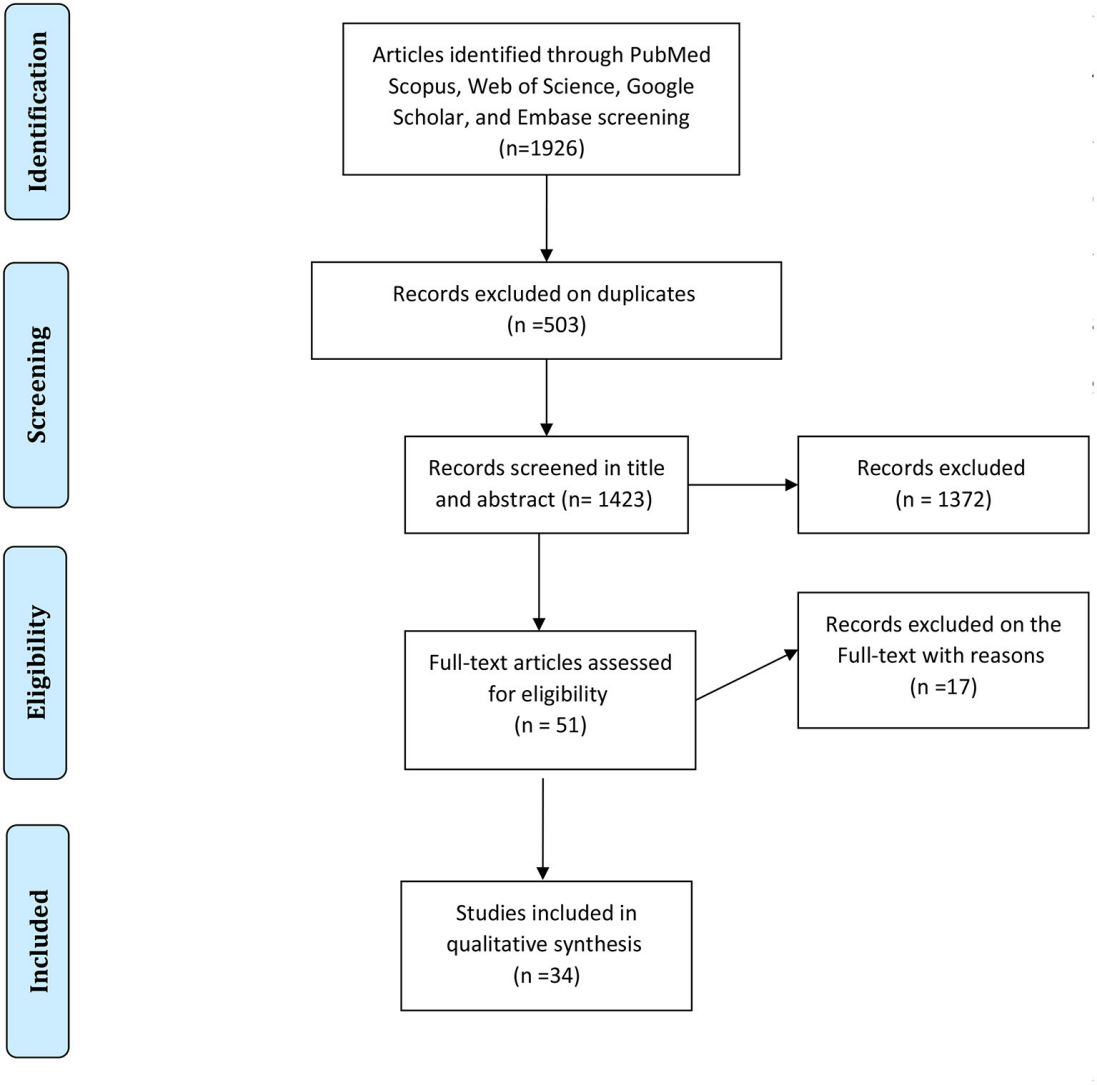
PRISMA flowchart showing the search and study selection strategy.

In each electronic database, various combinations of the following search terms were used: “Candidiasis, Oral,” and “HIV,” or “human immunodeficiency virus,” and “epidemiology.” Searching and collecting the relevant papers were done by two authors. The disagreements between the reviewers were solved with discussions between the two authors in a joint session, and, whether or not an agreement was not reached, a decision was made by a third author. At the end of the search, the collected articles were managed with EndNote X7.1 software (Thomson Reuters). A PRISMA flowchart showing the search and study selection strategy is presented in Figure 1.

### Inclusion and Exclusion Criteria

The inclusion criteria of the present research consisted of all original articles in the English language reporting the prevalence of oral *Candida* colonization or infection in HIV-infected children from the age of 0–18 years. Oral candidiasis can include both forms of oral *Candida* colonization and oral *Candida* infection. *Candida* colonization was defined as the isolation of ≥50 *Candida colonies from* the oral cavity without oral lesions (20). *Candida* infection included several lesions such as pseudomembranous, erythematous, and angularchelitis that most are caused in immunosuppressive patients. The most discrete lesion represents a conversion from benign colonization to pathological overgrowth (21).

In all included studies the diagnosis of HIV should be performed according to the World Health Organization (WHO) 1997's criteria. Exclusion criteria were: review articles, case reports, randomized controlled trials, letters, workshop reports, and case series without original data. We also excluded studies if they were not contained details of oral *Candida* species, done in HIV-infected patients older than 18 years of age, do not provide any information about the participant's age, published in a language other than English, investigated only a single pathogen of OC, discussed oral *Candida* carriage (C*andida* oral flora/oral *Candida* occurrence), reported sporadic OC, and were not contained patterns of clinical presentations associated with oral *Candida* colonization or infection.

### Data Extraction and Quality Assessment

The primary citations obtained during the database survey were recorded in a text file according to their topics and abstracts. Following an initial screening, especially eligible records were selected for full-text download. The final eligibility and inclusion criteria for the downloaded full texts were appraised by 2 separate investigators at three levels: title, abstract, and full text. Afterward, 2 authors extracted the requisite data. Authors have independently assessed the risk of bias in the included studies, according to the criteria from the modified STROBE checklist, as a validated method for assessing the quality of observational and case-control studies (22). The instrument used a system to evaluate cross-sectional studies based on some criteria: title and abstract, background, objectives, study design, setting, participants, variables, data sources, and measurements, bias, study size, quantitative variables, statistical methods, participants' result, descriptive data result, outcome data and main results, other analyses, key results, limitations, interpretation, and generalizability (22).

For each domain, the following description was used for management of the risk of bias: “Yes,” “No,” and “Unclear.” We graded the quality of included studies and risk of bias, using grading 1 for “yes” and 0 for “no and unclear,” and disagreement was resolved by discussion.

All included articles were prepared for data extraction by a pre-prepared checklist in Microsoft Excel spreadsheets. The checklist included the first author last name, year of publication, study period, the location of the study (country and continent), type of study, the number of HIV-infected pediatrics (sample size), age of participants, the prevalence percentage of oral *Candida* colonization and/or infection, gender distribution among patients with oral *Candida* colonization and/or infection, method for *Candida* species identification, the number of patients undergoing antiretroviral therapy (ART) or HAART, treatment status, the main etiologic agent of oral *Candida* colonization/infection, the predominant manifestations, and the commonest age group related to oral *Candida* colonization and/or infection. Regarding oral manifestations of *Candida* colonization and/or infection, we focused on the most significant ones including pseudomembranous, erythematous, and angular cheilitis, which were reported in the updated classification of HIV-related oral diseases (23). When the reported data were insufficient or in the case of articles whose full text was not available, we contacted the corresponding by email to request additional information or full text. After three emails with a week's interval, these studies were excluded.

### Statistical Analyses

All analyses were performed using SPSS software (IBM SPSS Statistics for Windows, Version 21.0, IBM Corp, Armonk, NY, USA). We used Random Effect Model for combining the effect size measures. The tau-squared (τ^2^ or Tau^2^) and *I*^2^ indexes were used to measure the heterogeneity in the results of studies. Studies with an *I*^2^ index 0–40%, 30–60%, 50–90%, 75–100%, categorized as might not be important, moderate heterogeneity, substantial heterogeneity, and considerable heterogeneity respectively (24). The publication bias was evaluated using Egger test (25). The results of the meta-analysis were visualized as a forest plot representing the prevalence estimates of each study. Statistical significance was defined as a *P* < 5%.

## Results

The first search identified a total of 1,926 records (212 in PubMed, 902 in Scopus, 193 in Embase, and 619 in Web of Science). After the removal of duplicates, 1,423 papers remained. Totally, 1,372 articles were not considered relevant after the title and abstract screening. Finally, 51 studies that met the inclusion criteria were reviewed in-depth and 34 study papers (3, 11, 26–56) were found to be eligible for meta-analysis. The detailed information of the 34 included studies is summarized in Table 1.

**Table 1:** The detailed information presented in the 23 included studies.

**Row**	**First author **	** Year of publication**	**Study period **	**Continent**	**Country**	**Type of study**	**Number of included HIV-infected pediatrics **	**Age of participants (years)**	**Prevalence%**		**HIV^**+**^ patients reciving antiviral therapies (%) **	**Treatment status**	**The main etiologic agent**		**Gender**		**Predominant clinical patterns of OC **	**Diagnostic methods**	**The commonest age group (years) **	**Study quality**	**References**
									**Coloni-zation**	**Infection**			***C. albicans***	**NAC species**	**Female**	**Male**				
1	Adejuyigbe E	2006	2005–2006	Africa	Nigeria	Cross–sectional	39	1.5–12	–	43.6%	0%	NA	NA	NA	–	–	Pseudo- membraneous	Clinical examination	NA	Low	(26)
2	Chen JW	2003	2000	Europe	Romania	Cross–sectional	104	NA	–	44%	44%	Under treatment	NA	NA	–	–	Angular cheilitis	Clinical examination	NA	Moderate	(27)
3	Domaneschi C	2011	2010	South America	Brazil	Cross–sectional	124	NA	62%	6%	100%	Under treatment	70%	30%	–	–	NA	Laboratory methods (KOH, xylose, and trehalose assimilation test, germ–tube test, thermotolerance test, CHROMagar culture)	NA	High	(28)
4	Gaitán–Cepeda L	2002	1999	North America	Mexico	Cross–sectional	48	NA	–	21%	0%	NA	NA	NA	5	5	Erythematous	Clinical examination + Laboratory methods (KOH, SDA culture)	NA	High	(29)
5	Gaitán–Cepeda L	2010	2005–2007	North America	Mexico	Cross–sectional	87	NA	–	21%	100%	Under treatment	NA	NA	–	–	Angular cheilitis	Clinical examination + Laboratory methods (KOH, SC culture)	NA	Low	(30)
6	Khongkunthian P	2001	2000	Asia	Thailand	Cross-sectional	45	NA	–	42.22%	33.3%	Under treatment	NA	NA	–	–	Erythematous	Clinical examination	N A	High	(31)
7	Meless D	2014	2011	Africa	Côte d'Ivoire, Mali and Sénégal	Cross–sectional	420	NA	–	4.8%	100%	Under treatment	NA	NA	–	–	Pseudo- membraneous	Clinical examination	NA	High	(32)
8	Olufemi Olaniyi T	2005	2004	Africa	Nigeria	Cross-sectional	36	1.5–18	–	45%	38.9%	Under treatment	NA	NA	–	–	Angularchelitis	Clinical examination	NA	Moderate	(33)
9	Pomarico L	2009	2009	South America	Brazil	Cross-sectional	65	NA	–	17%	76%	Under treatment	94%	6%	NA	NA	NA	Laboratory methods (SDA culture, CHROMagar culture, API 20C kit)	NA	High	(34)
10	Pongsiriwet S	2003	2002	Asia	Thailand	Cross–sectional	40	NA	–	45%	12.5%	Under treatment	93%	7%	NA	NA	Pseudo- membraneous	Clinical examination + Laboratory methods (SDA culture, germ tube test, CMA culture, hydrolysis of urea, sugar assimilation, and fermentation test)	NA	High	(35)
11	Santos LC	2001	1996	South America	Brazil	Cross–sectional	80	NA	–	22.5%	NA	NA	NA	NA	NA	NA	Pseudo- membraneous	Clinical examination	NA	High	(36)
12	Vaseliu N	2005	1998–2001	Europe	Romania	Cross-sectional	238	NA	–	11%	50%	Under treatment	NA	NA	NA	NA	Erythematous, Angular cheilitis	Clinical examination	NA	High	(37)
13	Gallottini Magalhães M	2002	2002	South America	Brazil	Cross-sectional	38	NA	–	36.8%	79%	Under treatment	NA	NA	NA	NA	Angularchelitis	Clinical examination + Laboratory methods (KOH, biopsies, cytological examinations, culture) + Radiography	NA	Moderate	(38)
14	Naidoo S	2004	2000–2001	Africa	South Africa	Cross–sectional	169	NA	–	55%	0%	NA	NA	NA	76	92	Pseudo- membraneous	Clinical examination	NA	High	(39)
15	Nabbanja J	2012	2007–2008	Africa	Uganda	Cross-sectional	368	1.5–18	–	60.8%	67.4%	Under treatment	NA	NA	NA	NA	Pseudo- membraneous	Clinical examination	NA	High	(40)
16	Mensana MP	2018	2017	Asia	Indonesia	Cross–sectional	28	NA	–	57.14%	42.85%	Under treatment	NA	NA	NA	NA	Angular cheilitis	Clinical examination + Laboratory methods (SDA culture, CHROMagar culture, hydrolysis of urea, carbohydrate fermentation tests, thermotolerance test, slide culture)	NA	Moderate	(3)
17	Shiboski CH	2001	1996–1999	NA	NA	Cross–sectional	294	13–19	–	6%	46%	Under treatment	NA	NA	NA	NA	Pseudo- membraneous	Clinical examination	NA	High	(41)
18	Oyedeji OA	2015	2013	Africa	Nigeria	Cross-sectional	58	NA	–	17.2%	63.7%	Under treatment	NA	NA	24	34	Angular cheilitis	Clinical examination	NA	Low	(42)
19	Adebola AR	2012	2005–2006	Africa	Nigeria	Cross-sectional	105	0–14	–	79.1%	61.95	Under treatment	NA	NA	NA	NA	Angular cheilitis	Clinical examination	NA	Low	(43)
20	Rwenyonyi CM	2011	2009	Africa	Uganda	Cross-sectional	237	1.5–12	–	28.3	49.7%	Under treatment	NA	NA	NA	NA	Pseudo- membraneous	Clinical examination	NA	High	(44)
21	Fonseca R	2000		South America	Brazil	Cross-sectional	51	NA	–	36.5%	NA	NA	NA	NA	NA	NA	Pseudo- membraneous	Clinical examination + Laboratory methods (Culture)	NA	High	(45)
22	Ramos-Gomez FJ	2000	1992	North America	California	Cross-sectional	40	NA	–	35%	NA	NA	NA	NA	NA	NA	Erythematous	Clinical examination	NA	Low	(46)
23	Ranganthan A	2010	2004–2005	Asia	India	Cross-sectional	212	NA	–	56.1%	0%	NA	NA	NA	NA	NA	Pseudo- membraneous	Clinical examination + Laboratory methods (SDA culture, germ tube test)	NA	Moderate	(47)
24	Sales-Peres SHC	2012	NA	Africa	Mozambique	Cross-sectional	90	NA	–	5.5%	81%	Under treatment	NA	NA	NA	NA	Angular cheilitis	Clinical examination	NA	Moderate	(48)
25	Ponnam SR	2012	NA	Asia	India	Cross-sectional	190	NA	–	19%	50%	Under treatment	NA	NA	NA	NA	Pseudo- membraneous	Clinical examination	NA	Low	(49)
26	de Aguiar Ribeiro A	2012	2010-2011	south America	Brazil	Cross-sectional	57	NA	–	15.78	100%	Under treatment	NA	NA	NA	NA	Angular cheilitis	Clinical examination	NA	Low	(50)
27	de Aguiar Ribeiro A	2015	2014–2015	south America	Brazil	Cross-sectional	111	NA	–	1.8%	87.3%	Under treatment	NA	NA	NA	NA	NA	Clinical examination	NA	Low	(51)
28	Baghirath PV	2013	2012	Asia	India	Cross-sectional	100	5–12	–	23%	50%	Under treatment	NA	NA	NA	NA	NA	Clinical examination	NA	Low	(52)
29	Kumar RK	2013	NA	Asia	India	Cross-sectional	326	1–14	–	20.86%	NA	NA	NA	NA	NA	NA	NA	Clinical examination	NA	High	(11)
30	Divakar DD	2015	2014	Asia	India	Cross-sectional	117	NA	–	15.3%	53%	Under treatment	NA	NA	NA	NA	NA	Clinical examination	NA	Low	(53)
31	Sánchez-Vargas LO	2005	2002–2005	North America	Mexico	Cross–sectional	60	NA	–	11.7%	83.3%	Under treatment	90%	10%	NA	NA	Erythematous	Clinical examination + Laboratory methods (SDA culture, germ tube test, CMA culture, indirect immunofluorescence assay, thermotolerance, test, chlamydoconidia production on Casein agar, API 20C kit)	NA	High	(54)
32	Portela MB	2004	NA	South America	Brazil	Cross–sectional	52	NA	–	43%	86.5%	Under treatment	74.2%	25.8%	NA	NA	NA	Clinical examination + Laboratory methods (CHROMagar, morphological and biochemical characteristics)	NA	High	(55)
33	Gallottini Magalhães M	2001	NA	South America	Brazil	Cross-sectional	38	NA	–	36.8%	80%	Under treatment	NA	NA	N	NA	Angular cheilitis	Clinical examination + Laboratory methods (biopsies, cytological examinations, culture) + radiography	NA	Moderate	(38)
34	Kolisa YM	2019	NA	Africa	South Africa	Cross-sectional	407	10-18	–	2.45%	100%	Under treatment	NA	NA	NA	NA	Pseudomembranous	Clinical examination	NA	High	(56)

*C. albicans, Candida albicans, NAC species, non-albicans Candida species; OC, oral candidiasis; CMA, corn meal agar; NA, not available; KOH, potassium hydroxide; SDA, sabouraud dextrose agar; SC, Sabouraud Chloramphenicol agar*.

**Figure 2:**
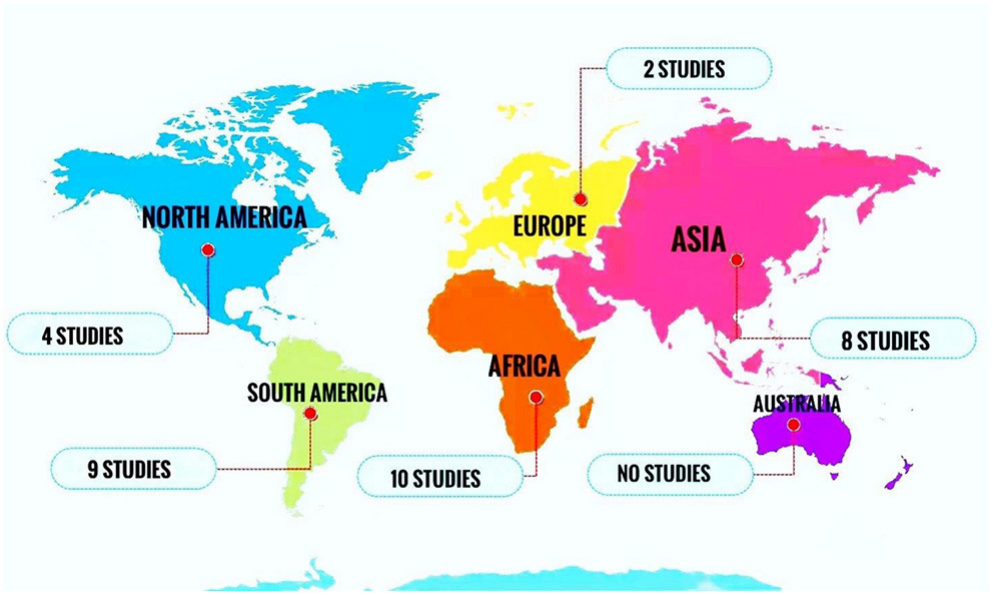
Number of studies reporting the prevalence of oral *Candida* colonization or infection in HIV-infected pediatrics in different continents.

All included papers were published between 2000 and 2019. The majority of the studies were conducted in Africa (*n* = 10), South America (*n* = 9), and Asia (*n* = 8) (Figure 2).

**Figure 3:**
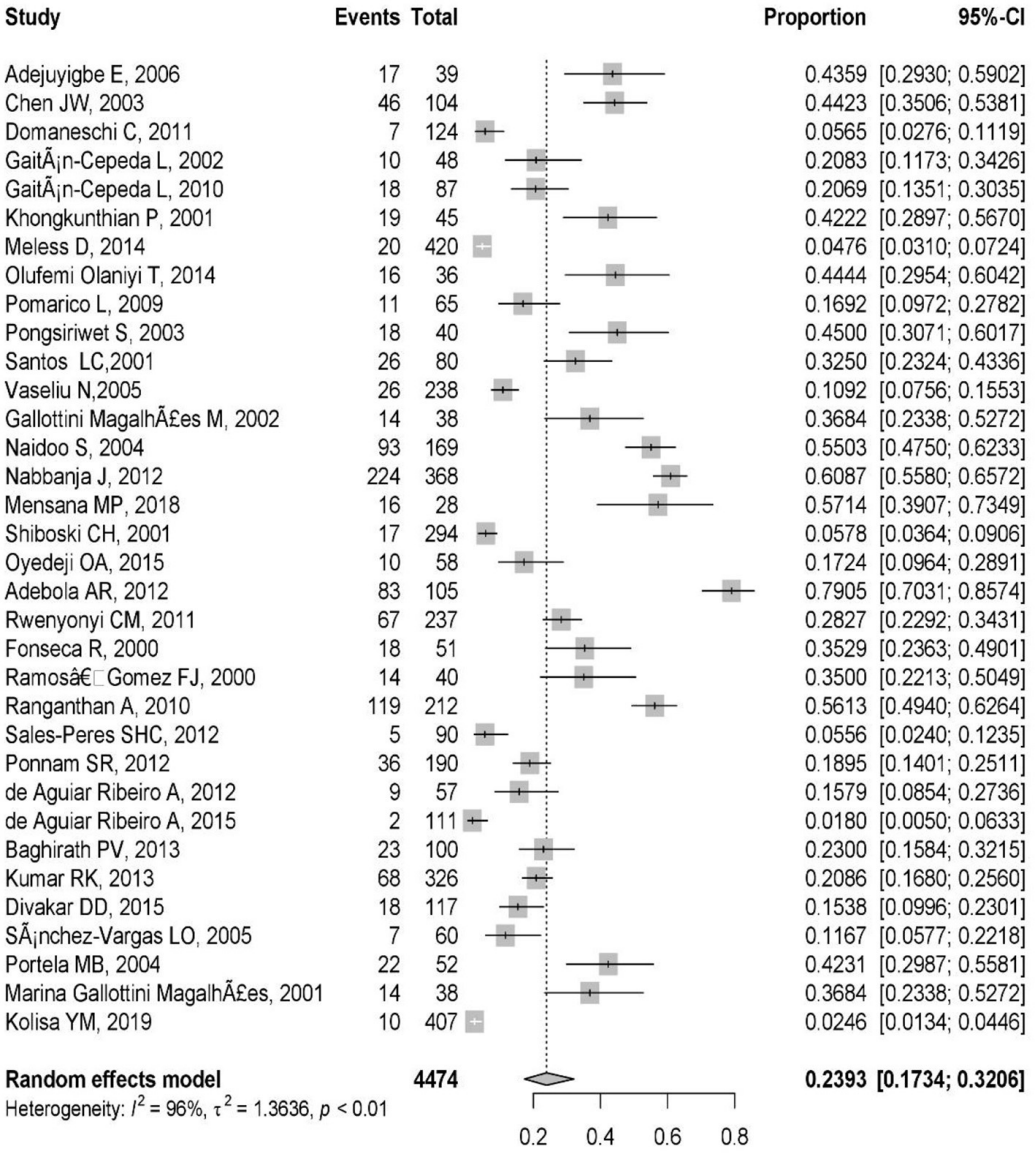
Forest plot of the prevalence of OC in HIV-infected pediatrics across the world.

These included studies consisted of a total of 4,474 HIV-infected pediatric patients from 12 different countries. The country with the highest number of studies was Brazil (9/34). The study with the smallest population sample was the one conducted by Mensana et al. (3) (28 patients), while the study by Meless et al. (32), with 420 patients, was the one with the largest sample. The majority of the articles (*n* = 33; 97%) reported OC, and one (3%) presented both OC and oral *Candida* colonization (Table 1). The prevalence of OC among HIV-infected pediatrics was ranged from 1.8% (95% CI: 0.5–6.3%) in Brazil to 79.1% (95% CI: 70.3–85.7%) in Nigeria. The pooled prevalence of OC among HIV-infected pediatrics was 23.9% (95% CI 17.3–32.0%) (Figure 3).

The high obtained percentage of *I*^2^ (*I*^2^ = 96%, *P* < 0.01) and τ^2^ (τ^2^ = 1.36, *P* < 0.01) represent the high variation and notable heterogeneity among the extracted studies (Figure 3). The Egger test results was statistically significant (*p* = 0.01) that shows there is publication bias.

In the majority of the included articles (*n* = 12; 35.29%) pseudomembranous candidiasis was reported as the most prevalent form of OC in HIV-infected pediatrics followed by angular cheilitis (*n* = 10, 29.41%), and erythematous candidiasis (*n* = 4, 11.77%). Also, a single article (*n* = 1, 2.94%) in Romania reported both erythematous candidiasis and angular cheilitis as the most prevalent form of OC (Table 1).

The prevalence of pseudomembranous candidiasis ranged from 1.7% in South Africa (56) to 72.2% in and Brazil (36). Also, the prevalence of angular cheilitis was highest in Indonesia, at 46.6% (3). Furthermore, the prevalence of erythematous candidiasis was highest in Thailand (35) at 55.5%.

Five articles reported the etiologic agents of OC among HIV-infected pediatric, and in all of them (*n* = 5, 100%) *Candida albicans* was the predominant etiologic agent (Table 1).

In the 34 reviewed studies clinical examination and/or laboratory techniques were used for the diagnosis of OC. In studies in which the diagnosis of OC carried out according to clinical examination, the evaluation of the oral health status of patients had been performed by an oral physician. The laboratory techniques used in these studies included: morphological identification (10% KOH, biopsies, cytological smears, and chlamydoconidia production on corn meal agar and Casein agar, slide culture, and immunofluorescence assay), culture method [sabouraud dextrose agar (SDA), sabouraud chloramphenicol agar (SC)], methods based on chromogenic substrates (CHROMagar), physiological methods (germ tube, thermotolerance test at 42–45°C, sugar assimilation and fermentation, hydrolysis of urea), and commercial identification kits (API 20C) (Table 1).

No clear data of the age and gender distribution regarding OC among HIV-infected pediatrics were given in the studies included in the meta-analysis (Table 1).

Thirty articles involving 4,057 individuals provided data on HIV treatment status. Among the 4,057 individuals, 468 (11.53%) did not receive HAART or ART. The data from 11 articles (28, 33, 43, 44, 48, 49, 51–54, 56) demonstrated that HAART or ART were significantly associated with reduction in oral *Candida* colonization or infection, while others showed the opposite relationship (12, 16, 32, 34, 41, 42, 55), or did not report any statistical data (11, 14, 26, 35–40, 47, 50).

## Discussion

Our results showed that most of the examined children came from African and South American countries. This confirmed the fact that these countries are those in which HIV is more widespread, especially among the pediatric population. Actually, Africa continent countries are home to about 15.2 percent of the world's population and more than two-thirds of the total HIV-infected patients worldwide are Africans (56). On the other hand, vertical transmission of HIV continues to be a largely uncontrolled problem in African countries where 95% of HIV-infected pregnant women live (57). Also, the results of four surveys in South America showed that although several countries in this continent have shown impressive declines in HIV incidence, the number of new HIV infections in the region increased and the main reason was that about 25% of 15–19-year-olds did not know how to protect themselves against HIV (58). Also, the country with the highest number of studies was Brazil. This finding could be explained by the tradition of dentistry research in this country.

The pooled prevalence of OC among HIV-infected pediatrics was 23.9% (95% CI 17.3–32.0%) (Figure 2) which is similar to the prevalence of OC in HIV-infected adult patients reported in Brazil (59, 60), Nepal (61), and United States (62). It was different from that reported in Uganda (63) and Indonesia (64) as they reported a prevalence of 70 and 66.7% for OC among HIV-infected adult patients. It should be noted, until now there is no comprehensive meta-analysis discussing the prevalence of OC in HIV-infected adults in the world, so the pooled prevalence related to it is unavailable. Our results showed that the prevalence of OC among HIV-infected pediatrics was ranged from 1.8% (95% CI: 0.5–6.3%) in Brazil (43) to 79.1% (95% CI: 70.3–85.7%) in Nigeria (51). Universal access to antiretroviral treatment in Brazil may explain the low incidence of OC among HIV-infected Brazilian pediatrics and difficult access to treatment may be the main reason for the high prevalence of OC observed in Nigerian HIV-infected pediatrics, as well as in the study from Nigeria 61.9% of investigated HIV-infected pediatrics were under treatment with antiretroviral therapy (43), while in the study from Brazil 87.3% of included HIV-infected pediatrics were under treatment (51).

Also, the prevalence of OC among HIV-infected pediatrics represented a high variation and notable heterogeneity. This difference in the prevalence of the infection in different studies can be due to the differences in the methodology used for the collection and analysis of data (in fact, the procedures that were used for the diagnosis of oral lesions and for the execution of statistical analysis in most of the studies have been not reported in a detailed and precise way).

In the studies included in the meta-analysis, we could also observe that access to treatment for HIV-positive pediatrics is still difficult: Only in 5 studies (27, 29, 31, 38, 49), 100% of children underwent treatment (ART or HAART), while results of other studies included in the meta-analysis (3, 26, 30, 32–34, 36, 37, 39–43, 47, 48, 50–55) showed that some but not all participating pediatrics were on antiretroviral therapy at the beginning of the study. Also, different studies on the effect of antiretroviral therapy on OC among HIV-positive pediatrics showed conflicting results. While some authors reported a significant reduction in the prevalence of OC (27, 32, 38, 42, 43, 47, 48, 50–53), others did not observe any changes in the occurrence of HIV-related OC in children receiving antiretroviral therapy (12, 16, 31, 33, 40, 41, 54). The reasons for such different results are not fully understood. Some authors have associated these variations with differences in oral care habits, social and demographic factors, HIV-transmission mode, and types of co-infections, disease stage, and immune reconstitution (64–66). Nevertheless, one should emphasize that oral lesions may develop again due to HAART failure and multi-drug resistance (67).

The following list of medications was used in the treatment of HIV in studies included in the present investigation: Zidovudine (26, 48, 52, 53), Lamivudine (26, 42, 48, 52), Nevirapine (42, 48, 52), Stavudine (42, 48), and co-trimoxazole (43).

Pseudomembranous candidiasis was the predominant clinical pattern of OC among HIV-infected pediatrics. In accordance, the majority of studies conducted around the world agree that pseudomembranous candidiasis is the most common clinical pattern of OC among HIV-infected patients (7). A second form erythematous candidiasis and a third form, angular cheilitis, were also reported as the most common clinical pattern of OC among HIV-infected patients (7). These data are in agreement with what emerged from this meta-analysis.

According to the results of the present study, *Candida albicans* is still the most prevalent species causing OC in HIV-infected pediatric patients. The ability to grow in both yeast and hyphal forms, the production of secreted proteinase activity, and the ability to biofilm formation are important virulence factors related to this microorganism (68–70). On the other hand, clinical manifestations of oral candidiasis vary depending on the type of causative agent. Pseudomembranous candidiasis is common in oral infections due to *Candida albicans* (71).

The present study shows that the epidemiology of HIV-associated oral candidiasis in pediatrics has not been studied in many countries worldwide. Also, it indicates in many countries the diagnosis of oral candidiasis in HIV-infected pediatrics is made based on clinical signs and symptoms, and without laboratory testing. Due to the fact that different *Candida* species have various degrees of susceptibility to common antifungal agents and the increasing prevalence of infections caused by non-*C. albicans Candida*, there is a need for investigating the distribution of *Candida* species causing oral candidiasis in each country, and clinicians should be aware of the importance of laboratory testing in diagnostic decision making and know that the identification of the causative agents at the species level is essential for appropriate treatment (14, 15). In addition, the opportunistic *Candida* species existing as part of commensal microflora in humans are usually the etiological agents causing infections. Hence, isolates from various sources and patients of different climate regions may have different characters.

Among limitations to be noted in this study is that we did not attempt to identify studies from the “gray” literature, such as conference proceedings or research published in master's or PhD theses. Another limitation is that we did not include any articles published in languages other than English. Also, because the search of this study has been done worldwide and the number of preliminary studies for selection was high, we used date restriction filter to limit primary studies. Furthermore, there was evidence of statistical heterogeneity when calculating a pooled estimate of the prevalence of OC among HIV-infected pediatrics. As discussed above, we feel the most likely source of heterogeneity was the differences in the methodology used for the collection and analysis of data, because the procedures that were used for the diagnosis of oral lesions and for the execution of statistical analysis in most of the studies have been not reported in a detailed and precise way.

## Conclusion

The present systematic review and meta-analysis gives an insight into the scenario of OC related to HIV-infection among pediatrics in the two last decades and helps to provide a comprehensive overview on the most common oral manifestations in HIV-positive pediatrics. Given that this infection can lead to fatal complications such as candidemia, meningitis, endocarditis, esophagitis, and endophthalmitis in HIV-positive pediatrics whose immune systems are very weak taking preventive measures such as awareness of parents as well as monitoring and controlling of symptoms are essential. On the other hand, it is very important that physicians and dentists know the main clinical manifestations and associated factors of oral candidiasis among HIV-infected pediatrics, since it may be the first manifestation of a local or systemic disorder.

## Data Availability Statement

The original contributions presented in the study are included in the article/supplementary material, further inquiries can be directed to the corresponding author/s.

## Author Contributions

ZR, MS, and ES: conceptualization, supervision, and project administration. ZR: writing original draft. ES, YS, SH, and DR: data curation, methodology, and validation. YS: data analysis. ZR, ES, and YS: writing-review and editing. All authors discussed the results, contributed to the final manuscript, and approved the submitted version.

## Conflict of Interest

The authors declare that the research was conducted in the absence of any commercial or financial relationships that could be construed as a potential conflict of interest.

## Publisher's Note

All claims expressed in this article are solely those of the authors and do not necessarily represent those of their affiliated organizations, or those of the publisher, the editors and the reviewers. Any product that may be evaluated in this article, or claim that may be made by its manufacturer, is not guaranteed or endorsed by the publisher.
